# Lessons learned on Zika virus vectors

**DOI:** 10.1371/journal.pntd.0005511

**Published:** 2017-06-15

**Authors:** Ricardo Lourenço-de-Oliveira, Anna-Bella Failloux

**Affiliations:** 1 Laboratório de Mosquitos Transmissores de Hematozoários, Instituto Oswaldo Cruz, Rio de Janeiro, Brazil; 2 Institut Pasteur, Arboviruses and Insect Vectors, Paris, France; University of Queensland, AUSTRALIA

Today, a wide belt of the Earth is receptive or vulnerable to epidemics of mosquito-borne diseases, not only in the intertropical zone in the American, African, and Asian continents but also in some European and Pacific regions. Billions of (usually low-income) people who are immunologically naïve to potentially emergent or re-emergent arboviruses live in some of these areas.

The most recent examples of disasters caused by mosquito-borne arboviruses are the 2015–2016 re-emergences of urban yellow fever (YF) in Angola, which reached the Democratic Republic of Congo, with viremic people dispersing to densely populated regions in Asia, such as China [1], and the global emergence and spread of chikungunya and Zika viruses in the Pacific region and the Americas [2,3].

Since vaccines are unavailable or inadequately supplied and drugs are inefficient in the treatment of arbovirus infections, prevention and control of such infections must rely on the fight against the insect vectors of the viruses. The implementation of efficient and effective vector control programs depends on specific knowledge of vector competence, identity, bioecology, and behavior. To appraise the likely role of a mosquito species in transmitting an arbovirus by assessing vector competence may help in determining the risk of arbovirus transmission and spread and, more importantly, in structuring efficient vector control by targeting the correct vector species.

The 2015–2016 Zika virus (ZIKV) outbreak in the Americas and the Caribbean is an unparalleled epidemiological situation, and it remains an alarming international public health threat. Indeed, after its first emergence in Yap Island in Micronesia, outside its traditional region in Africa and Asia, this virus rapidly spread to 67 countries or territories and infected more than 2 million people, causing symptoms ranging from mild to severe [3, 4]. Although sexual and other interhuman ZIKV contaminations have been confirmed, the primary route of viral transmission is accomplished through the bite of an infected *Aedes* mosquito. Mosquito-borne ZIKV transmission has only been reported in *Aedes* (*Stegomyia*) infested territories, and *Ae*. (*Stg*.) *aegypti* has been considered its main vector across the world [3,4,5].

Studies of vector competence for ZIKV were very limited until the most recent emergences [3]. From 2015 onwards, the number of peer-reviewed vector competence studies rapidly increased, especially due to the hypothesis that other non-*Stegomyia* mosquitoes could be primary ZIKV vectors. Due to their human-orientated feeding behavior and abundance indoors, *Culex* mosquitoes of the Pipiens Assemblage came momentarily under suspicion and increased attention. As a result, 18 populations from all 5 continents were independently tested in 10 laboratories across the world [5–14] (S1 Table). Briefly, 8 *Culex pipiens* and 10 *Cx*. *quinquefasciatus* populations have proved to be incompetent to transmit 10 isolates of the circulating Asian genotype of ZIKV and 2 of the African genotype, including the prototype of ZIKV, even when challenged with high viral loads fed either directly on viremic animals or on artificial meals and incubated under various conditions (see S1 Table). In contrast, *Stegomyia* mosquitoes were competent to transmit ZIKV when simultaneously challenged with the same isolates and viral loads [5,7,9,11,12,14]. Only 1 discordant study described the detection of RNA fragments compatible with ZIKV in the saliva of orally challenged *Cx*. *quinquefasciatus* mosquitoes [6]. Notably, infectious ZIKV particles have never been detected in saliva expectorated by *Cx*. *pipiens* or *Cx*. *quinquefasciatus* mosquitoes either orally exposed or intrathoracically inoculated with ZIKV [5, 7–14]. Moreover, *Culex* mosquitoes essentially do not even meet the basic requirements that allow a potential transmission, i.e., the persistence of infection followed by viral dissemination to secondary tissues outside the midgut [5, 7–9, 11–13]. In reality, *Culex* mosquitoes infrequently become infected, or at most, they are minimally infected, but viral dissemination is consistently not achieved [5, 7–14].

Therefore, these results provide enough consistent scientific evidence to conclude that domestic *Culex* mosquitoes cannot be considered ZIKV vectors. Consequently, there is no reason to consider *Culex* as target species to halt the ZIKV epidemics and spread. It is past the time to stop controversy and focus our efforts on research on several still poorly understood aspects of ZIKV dynamics of transmission and to propose more accurate surveillance methods and adapted control measures against *Ae*. *aegypti*. Designing combinations of efficient control measures against this mosquito would concurrently mitigate transmission of dengue, YF, and chikungunya viruses, which share the same primary vector. Let’s join our efforts to face the real challenges. Other arboviruses are already on the starting blocks, as recently exemplified by Mayaro virus [15].

## Supporting information

S1 Table Summary of peer-reviewed studies assessing vector competence to Zika virus in domestic species of *Culex* belonging to the Pipiens Assemblage. (DOCX)
