# Supplementary material for: Lessons learned on Zika virus vectors
Source: PLoS Negl Trop Dis. 2017 Jun 15;11(6):e0005511. doi: 10.1371/journal.pntd.0005511 (PMC5472277; doi:10.1371/journal.pntd.0005511)
Supplement: S1 Table — (DOCX) [file pntd.0005511.s001.docx]

Summary of peer-reviewed studies assessing vector competence to Zika virus in domestic species of *Culex* belonging to the Pipiens Assemblage.

| **Mosquitoes** | | | **Virus** | **Experimental methods** | | | **Vector competence parameters (rates in %)** | | | **Comparison with vector competence in *Ae. aegypti* (14 dpi)** | **Reference** |
| --- | --- | --- | --- | --- | --- | --- | --- | --- | --- | --- | --- |
| **Species** | **Origin** | **Used generation** | **Origin and strain*^a^***  **(viral load in the blood meal)** | **Incubation conditions** | **Number of mosquito (N) examined each day after exposure (dpi)** | **Method for assessing viral infection** | **IR** | **DE** | **TE** | **Origin, generation and rates** |  |
| *Cx. pipiens* | Roma, Italy | Colony started in 2015 | French Polynesia, 2013  ZIKV H/PF/2013  6.46 log_10_ PFU/ml | 26+1˚C  14L:10D light cycle | N=10  dpi= 0, 3, 7, 10, 14, 20, 24 | RT-qPCR | 10  [3dpi] | 0 | 0 | Mexico (colony started 1998)  IR= 50%  DR= 50%  TE= 37.5% 14dpi | 7 (Boccoloni et al. 2016) |
| *Cx. pipiens* | Anderson, CA, USA | Colony, since 2014 F15 | Puerto Rico, 2015  ZIKV PRVABC59  6.52 logTCID_50_/mL | 28˚C 16L:8D light cycle | N= 17-34  dpi= 0, 7, 14 | titration on Vero cells culture  +  RT-PCR | 0 | 0 | 0 | ND | 8 (Huang et al. 2016) |
| *Cx. pipiens* | Ewing, Mercer, NJ, USA | Colony, since 2015  F7 | Puerto Rico, 2015  PRVABC59  7.52 logTCID_50_/mL |  | N=8-20  dpi= 0, 7, 14 |  | 0 | 0 | 0 | ND | 8  (Huang et al. 2016) |
| *Cx. quinquefasciatus* | Vero Beach, FL, USA | Colony, since 2015  F7 | Puerto Rico, 2015  PRVABC59  6.95 logTCID_50_/mL |  | N= 20-30  dpi= 0, 7, 14 |  | 0 | 0 | 0 | ND | 8  (Huang et al. 2016) |
| *Cx. pipiens* | Iowa, USA | Colony started in 2002 | Puerto Rico, 2015  PRVABC59  4.74, 6.02 and 6.83 log_10_PFU/mL | NI  N= 10-30 | dpi= 14 | Titration on Vero cells  (plaque assays) | 0 | 0 | 0 | Black-eyed Liverpool strain (colony)  IR= 100%  DR= 71%  TE=24% 14dpi | 9 (Aliota et al. 2016) |
| *Cx. quinquefasciatus* | San Joaquin Valley, California, USA | Colony, since 1950 | New Caledonia, 2014  NC-2014–5132  10^7.2^ PFU/mL | 28˚C  16L:8D light cycle | N= 40-48  dpi= 3, 7, 14, 21 | Titration on Vero cells  (plaque assays) | 0, 2.1, 17.0 and 12.5  [3, 7,14 and 21dpi] | 0, 0, 2.4 and 7.5  [3, 7,14 and 21dpi] | 0 | ND | 10 (Amraui et al. 2016) |
| *Cx. pipiens* | Tabarka,  Tunisia | Colony, since 2010 | New Caledonia, 2014  NC-2014–5132  10^7.2^ PFU/mL | 28˚C  16L:8D light cycle | N= 40-48  dpi= 3, 7, 14, 21 | Titration on Vero cells  (plaque assays) | 2.0, 6.3, 0 and 13.0  [3, 7,14 and 21dpi] | 0 | 0 | ND | 10 (Amraui et al. 2016) |
| *Cx. quinquefasciatus* | Four populations [COP, MAN, TRI and JAC] from Rio de Janeiro, Brazil | F1 (except for TRI, a colony started in 2010) | Rio de Janeiro, Brazil  RIO U-1 and RIO 17-S  10^6^ PFU/mL | 28˚C  12L:12D light cycle | N= 4-30  dpi= 7, 14 21 | Titration on Vero cells  (plaque assays)  +  RT-qPCR | 3.3  [TRI, 14dpi]  6.2  [MAN, 14dpi] | 0 | 0 | Urca and Paquetá, Rio de Janeiro, Brazil (F1 and F2)  IR= 92-100%  DR= 85-97%  TE= 60.6 – 93.3%  14 dpi | 5 (Fernandes et al. 2016) |
| *Cx. quinquefasciatus* | Sebring, Florida, USA | Colony, since 1988 | Puerto Rico, 2015  PRVABC59  5.0x10^6^  and  1.6x10^7^ PFU/mL | 28˚C | N= 144  dpi= 7, 14 | Titration on Vero cells  (plaque assays) | 0.3 and 0  [7 and 14 dpi] | 0 | 0 | Poza Rica, Mexico  (colony started in 2012) | 12 (Weger-Lucarelli et al. 2016) |
| *Cx. quinquefasciatus* | Houston, Texas, USA | F2 and colony | Cambodia, 2010 FSS13025; Mexico, 2015, MEX1–7 and MEX1-44; Senegal, 1985, DAKAR41525 and Puerto Rico, 2015  PRVABC59.  10^7^, 10^6^, 10^6^, 10^6^ and 10^7^FFU/mL, respectively | 27˚C | N= 5-26  dpi= 3,7,14 | Focus forming assays | 0 | 0 | 0 | NI | 13 (Hart et al. 2017) |
| *Cx. pipiens* | Pennsylvania, USA | Colony, since 2002 | Puerto Rico, 2015  ZIKV PRVABC59  5.0x10^6^  and  1.6x10^7^ PFU/mL | 28˚C | N= 48  dpi= 7, 14 | Titration on Vero cells  (plaque assays) | 0 | 0 | 0 | Poza Rica, Mexico  (colony started in 2012) | 12 (Weger-Lucarelli et al. 2016) |
| *Cx. quinquefasciatus* | Brisbane, Australia | F0 | Zika Forest, Uganda, 1947  MR 766  10^6.7 ± 0.2^ TCID_50_/mL | 28°C, 12L:12D light cycle | N= 30  dpi= 14 | TaqMan RT-PCR | 7  [14 dpi] | 0 | 0 | Townsville, Queensland, Australia, (colony started in 2015, F4)  IR=57%  DR= 71%  TR=27% 14dpi | 11 (Hall-Mendelin et al. 2016) |
| *Cx. pipiens* | Hamburg, Germany | F0 | USA (putative country of infection: Guatemala), 2016  FB-GWUH-2016  10^7^ PFU/mL | 18 and 27°C | N= ~35  Dpi= 14, 21 | Titration on Vero cells  (plaque assays)  +  RT-qPCR | 8, 47, 9 and 0  [14 and 21] | ND | 0 | Bayer Company (colony)  IR= 49-72%  TR= 0-45%  14 and 21 dpi | 14 (Heitnman et al. 2017) |
| *Cx. pipiens*  (biotype *molestus*) | Heidelberg, Germany | Colony since 2011 | USA (putative country of infection: Guatemala), 2016  FB-GWUH-2016  10^7^ PFU/mL | 18 and 27°C | N= ~35  Dpi= 14, 21 | Titration on Vero cells  (plaque assays)  +  RT-qPCR | 6, 29, 24, 32  [14 and 21] | ND | 0 | Bayer Company (colony)  IR= 49-72%  TR= 0-45%  14 and 21 dpi | 14 (Heitnman et al. 2017) |
| *Cx. quinquefasciatus* | Hainan, China | Colony since 2014 | Samoa,  SZ01  3 x 10^5^ PFU/mL | 29 + 1°C, 14L:10D light cycle | N= 10  dpi= 2, 4, 6, 8, 12, 16, 18 | RT-qPCR | 80, 20, ~40, 10, 10, ~50, and ~40  [2, 4, 6, 8, 12, 16 and 18 dpi] | 0, 10, 40, 40, 0, 20, and 20*^b^*  [2, 4, 6, 8, 12, 16 and 18 dpi] | 10, 20, 70, 90, 10, 10, ~20, and ~40  [2, 4, 6, 8, 12, 16 and 18 dpi] | ND | 6 (Guo et al. 2016) |

*a:* all *Culex* assays were conducted with ZIKV isolates belonging to the Asian Genotype, except for *Cx. quinquefasciatus* from Australia (Hall-Mendelin et al. 2016) and Texas, USA (Hart et al. 2017) , which were also challenged with the African Genotype; *b*: percentage of infected ovaries; IR: Infection rate refers to the proportion of mosquitoes with infected body (abdomen and thorax) among tested mosquitoes; DE: Dissemination efficiency corresponds to the proportion of mosquitoes with infected head, legs and/or wings among the initially tested mosquitoes; ND: Not Done; NI: Not Informed; PFU: plaque-forming units; TCID: tissue culture infectious dose; TE: Transmission efficiency represents the proportion of mosquitoes with infectious saliva among the initial number of mosquitoes tested.
